# Current European guidelines for management of arterial hypertension: Are they adequate for use in primary care? Modelling study based on the Norwegian HUNT 2 population

**DOI:** 10.1186/1471-2296-10-70

**Published:** 2009-10-30

**Authors:** Halfdan Petursson, Linn Getz, Johann A Sigurdsson, Irene Hetlevik

**Affiliations:** 1 Department of Family Medicine, University of Iceland, Solvangur Health Centre, IS-220 Hafnarfjördur, Iceland; 2 Research Unit of General Practice, Department of Public Health and General Practice, Norwegian University of Science and Technology (NTNU), Trondheim, Norway

## Abstract

**Background:**

Previous studies indicate that clinical guidelines using combined risk evaluation for cardiovascular diseases (CVD) may overestimate risk. The aim of this study was to model and discuss implementation of the current (2007) hypertension guidelines in a general Norwegian population.

**Methods:**

Implementation of the current *European Guidelines for the Management of Arterial Hypertension *was modelled on data from a cross-sectional, representative Norwegian population study (The Nord-Trøndelag Health Study 1995-97), comprising 65,028 adults, aged 20-89, of whom 51,066 (79%) were eligible for modelling.

**Results:**

Among individuals with blood pressure ≥120/80 mmHg, 93% (74% of the total, adult population) would need regular clinical attention and/or drug treatment, based on their total CVD risk profile. This translates into 296,624 follow-up visits/100,000 adults/year. In the Norwegian healthcare environment, 99 general practitioner (GP) positions would be required in the study region for this task alone. The number of GPs currently serving the adult population in the study area is 87 per 100,000 adults.

**Conclusion:**

The potential workload associated with the European hypertension guidelines could destabilise the healthcare system in Norway, one of the world's most long- and healthy-living nations, by international comparison. Large-scale, preventive medical enterprises can hardly be regarded as scientifically sound and ethically justifiable, unless issues of practical feasibility, sustainability and social determinants of health are considered.

## Background

The interest in preventive measures for cardiovascular diseases (CVD) has escalated in the last decades [1]. Apart from smoking and elevated cholesterol, hypertension has for the last fifty years been considered the most predictive CVD risk factor. The first international report highlighting the importance of blood pressure control was published in 1962 by the World Health Organisation (WHO) [2]. After this milestone publication several generations of clinical hypertension guidelines have followed on both sides of the Atlantic [3-13]. In 2003, the European Society of Hypertension (ESH) and the European Society of Cardiology (ESC) published their own guidelines on hypertension treatment, having until then endorsed the guidelines issued by the WHO and the International Society of Hypertension (ISH) [9]. The 2003 hypertension guidelines became the most quoted paper in the medical literature [13], and the guidelines were updated in 2007 [13].

For the last decade, combined CVD risk evaluation instruments have gained an important role in CVD prevention guidelines [8,12,14-16]. First prominent in the 1999 guidelines from the WHO/ISH [4], and followed by the 2003 [9] and 2007 [13] publications by the ESH/ESC, such estimates have also become central in hypertension guidelines. During the same time period, however, the threshold for intervention in relation to individual risk factors has also been lowered. The 2007 ESH/ESC guidelines also present a new risk factor, high pulse pressure (systolic minus diastolic blood pressure) in the elderly, in its combined risk model.

To be implementable in the everyday clinical setting, it is essential that guidelines harmonise with clinical and practical realities. Both the number of patients in need of treatment and the treatment goals should appear reasonable, both from a societal and a local clinical perspective. When the approach of guidelines to CVD risk identification and stratification changes, it is hard to foresee the consequences in terms of the population-at-risk and the clinical workload. One way to address this important topic would be to conduct modelling studies as an integral part of guideline development. Empirical modelling studies of clinical guidelines, however, are surprisingly rare. Some recent papers [17-20], including studies from our own group [21] have shown that the 2003 European Guidelines on CVD Prevention [8] significantly overestimated CVD risk in several European regions. Consequently, there is a strong argument for assessing the potential impact of new clinical guidelines.

The aim of the present study was to model the implications of the most recent European guidelines for the management of arterial hypertension [13] in a general Norwegian population. We primarily estimated the prevalence of individuals with unfavourable CVD risk levels according to the guidelines. Subsequently, the potential clinical workload and workforce associated with reaching recommended treatment goals in this group were calculated. We finally reflect upon the implications of our findings.

## Methods

Data from a large and renowned population study (the HUNT 2 Study, see ) [22] allowed us to calculate the proportion of the population with an unfavourable combination of risk factors, as defined by the 2007 guidelines [13]. Based on these figures, we estimated the number of follow-up visits needed to achieve the guidelines' recommended treatment goals. This number was again translated into the number of general practitioners (GP) potentially needed to carry out this work.

Norway is a country with a solid primary healthcare system, and every citizen is listed with a GP. Care is mostly delivered by the GPs and rarely by other trained staff, such as nurse practitioners. Our model was designed to fit into this context. In the following, we will present some essential details about the HUNT 2 data and our modelling of the clinical workload associated with the 2007 guidelines.

### The HUNT 2 population data

The Nord-Trøndelag Health Study 1995-97 (HUNT 2) has been described in detail elsewhere [22]. The overall participation rate in HUNT 2 was 76% among women and 67% among men. The HUNT 2 population has been considered representative of the total Norwegian population regarding demography, socio-economic factors, morbidity and mortality, including mortality from CVD [22].

Our model is based on data from all HUNT 2 participants aged 20-89 years, in total 65,028 individuals (30,447 males and 34,581 females), see Table 1.

**Table 1:** Participants in the study

**Age groups**	**Participants in HUNT-2**			**Eligible**		
		
	**Men**	**Women**	**Total**	**Men**	**Women**	**Total**
20-24	1761	2156	3917	1293	1085	2378
25-29	2163	2561	4724	1703	1202	2905
30-34	2579	2917	5496	2085	1362	3447
35-39	2820	3207	6027	2315	1645	3960
40-44	3161	3478	6639	2670	2140	4810
45-49	3334	3566	6900	2920	2520	5440
50-54	3064	3314	6378	2748	2631	5379
55-59	2333	2461	4794	2121	2086	4207
60-64	2113	2292	4405	1934	2057	3991
65-69	2232	2418	4650	2095	2249	4344
70-74	2134	2382	4516	1980	2240	4220
75-79	1594	2064	3658	1474	1942	3416
80-84	820	1231	2051	726	1127	1853
85-89	339	534	873	283	433	716
**Total**	30447	34581	65028	26347	24719	51066

Participants in the HUNT-2 (1995-7) study and those eligible for modelling in the present study according to age and gender.

Of these, 12,139 individuals (3,085 men and 9,054 women) had to be excluded because they had blood pressure levels below 120/80 mmHg (the 2007 guidelines do not address this group). Additionally, 1,015 men and 808 women had missing data regarding blood pressure or other factors of the six risk factors considered. In total, this rendered 51,066 HUNT 2 participants (79%) eligible for our modelling procedure. Among the 13,962 excluded participants, however, 788 (5.6%) did report established CVD, diabetes or receiving blood-pressure-lowering treatment. Our study thus underestimates the population-in-need-of-attention and associated workload at this point.

The participation rates in the HUNT 2 study were different in different age groups, with lower rates among the younger participants. When estimating the annual number of follow-up visits, this unequal participation rate was corrected for by age-standardising the HUNT 2 data with the 2007 age distribution in Nord-Trøndelag, which is similar to Norway in general [23,24]. This gives the younger age-groups, and hence the lower risk levels, increased weight in our calculations.

### Variables studied

**Figure 1:**
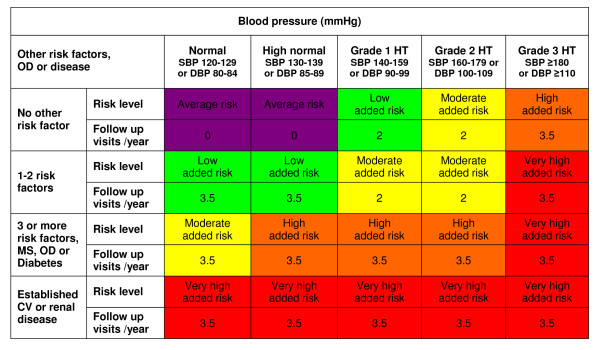
**Cardiovascular risk stratification chart with recommended follow-up frequency for each category**. A reconstruction of Figure 1 from the 2007 Guidelines for Management of Arterial Hypertension [13], with inserted recommendations regarding the number of follow-up visits per year in each risk category. Low, moderate, high and very high risk refer to the 10-year risk of a CV fatal or non-fatal event. The term 'added' indicates in all categories that risk is greater than average. The risk factors referred to in the left column are: age, smoking, dyslipidaemia, elevated fasting plasma glucose, abnormal glucose tolerance test, abdominal obesity, a family history of premature CVD and 'high pulse pressure in the elderly'. Abbreviations: SBP: systolic blood pressure; DBP: diastolic blood pressure; HT: hypertension. OD: subclinical organ damage; MS: metabolic syndrome.

**Figure 2:**
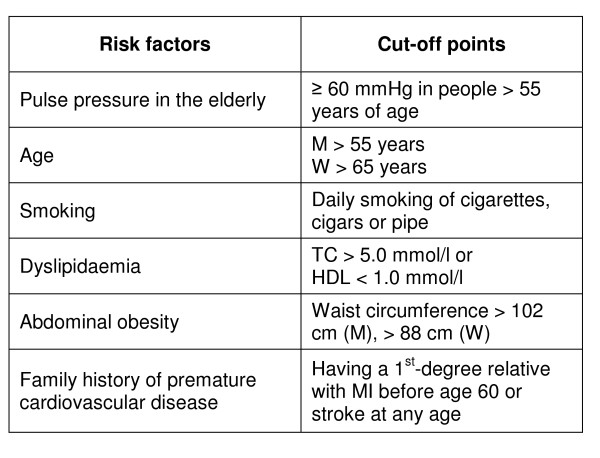
**Risk factors and cut-off points**. The risk factors and the cut-off points used in the present study, based on the 2007 Guidelines for the Management of Arterial Hypertension [13]. Abbreviations: M: men; W: women; TC: total cholesterol; MI: myocardial infarction.

The basis for our model is the definition and classification of blood pressure levels, as defined by the guidelines, see Figure 1. The determination of an individuals' risk level however also depends on the presence of other relevant risk factors. Figure 2 gives an overview of these, including the cut-off points applied in our modelling procedure.

In the present dataset, smoking was defined as daily smoking of cigarettes, cigars or a pipe. Family history of CVD was defined as 1^st^-degree relatives (parents, brothers and/or sisters) with myocardial infarction before age 60 or stroke at any age. Established CVD was defined as self-reported myocardial infarction, stroke or angina pectoris. Methods for measurement of blood pressure and body composition are described elsewhere [22].

Some risk factors listed in the guidelines had to be omitted from our model as they were not assessed in the HUNT 2 study. These were: abnormal glucose tolerance test, fasting plasma glucose, LDL-cholesterol and triglyceride levels (HUNT 2 participants were not fasting). People with renal disease and/or subclinical organ damage were not accounted for separately.

The 2007 guidelines give no details regarding the cut-off points for 'levels of pulse pressure (in the elderly)'. After reviewing the literature, we defined 'elderly' as above 55 years of age (the same definition as the guidelines used for age as an independent risk factor in men) and 'high' pulse pressure level as ≥60 mmHg [25-35].

### Estimation of clinical workload

Our estimates of the clinical workload related to each CVD risk category have been inserted in Figure 1. The number of follow-up visits are based on the guidelines' specific recommendations [13], when possible. As the follow-up frequency is not always accurately specified, we needed to make some interpretations, which we justify in detail below.

- Individuals at the lowest risk level (called 'average risk') are said to need no blood pressure intervention, and therefore we set the number of yearly visits to zero for this category.

- The guidelines' Box 22 ('Patients' follow-up', p. 1513) states that "Patients at low risk or with grade 1 hypertension may be seen every 6 months...". We therefore use 2 visits per year for these categories.

- The guidelines subsequently state that "Visits should be more frequent in high or very high risk patients. This is the case also in patients under non-pharmacological treatment alone due to the variable antihypertensive response and the low compliance with this intervention". We defined the term "more frequent" (than 2 visits per year) to mean 3-4 visits per year. Based on the above quote, we allocated an average of 3.5 visits per year for the categories 'high added risk,' 'very high added risk' and individuals with 'low added risk' who exhibit BP <140/90 under non-pharmacological surveillance due to the presence of other risk factors.

Since the choice of 3.5 visits per year on average for the most demanding follow-up categories can be discussed, we analysed our model's sensitivity to changes regarding this number. Alternative analyses based on 3.0 and 4.0 visits per year are also presented.

The guidelines recommend that "patients should be seen often (e.g., every 2 to 4 weeks)" [13] during the blood pressure drug titration phase. Our model however does *not *include visits needed to formally diagnose hypertension, nor the series of visits associated with initial drug titration. As we include only follow-up visits beyond that point, our model will underestimate workload.

As mentioned, the guidelines only address individuals with blood pressure levels of at least 120/80 mmHg, and people with lower blood pressure are excluded from this model, regardless of their medical history.

### Estimating the necessary primary care workforce

The prevalence of individuals assigned to each of the risk categories outlined in Figure 2 was calculated as a basis for analysis of clinical workforce needed.

In 2007 (January 1st), the Nord-Trøndelag County had 129,069 inhabitants. The population aged 20-89 accounts for about 72% of the total [23]. Nord-Trøndelag County was served by 112 GPs in 2007 [36]. This translates into 87 GPs per 100,000 inhabitants. This GP density is quite comparable to Norway as a whole (90 GPs per 100,000 inhabitants). We estimated the same number of GPs (87) to take care of every 100,000 adults (i.e., individuals eligible for our study). When calculating the medical workforce needed, we assumed that each GP in Nord-Trøndelag would conduct an average of 3000 consultations per year, which is equal to the Norwegian average [36].

### Statistics

The SPSS statistical package, version 15.0, was used for statistical frequency analyses.

### Ethical approval

The HUNT 2 survey in the Nord-Trøndelag health study was approved by the Norwegian Data Inspectorate and the regional committee for ethics in medical research.

## Results

**Figure 3:**
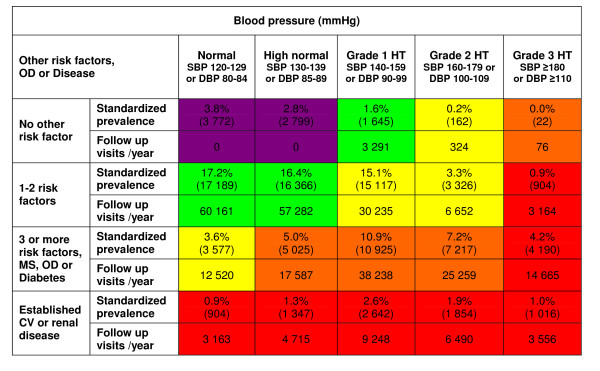
**Age-standardised prevalence of individuals in each risk category and associated number of follow-up visits**. Age-standardised prevalence for each risk category in relation to blood pressure levels (absolute numbers within brackets) as well as the calculated number of follow-up visits needed each year according to the 2007 Guidelines for the Management of Arterial Hypertension [13] per 100,000 adults, aged 20 to 89 in the HUNT 2 Study, Norway. Abbreviations: OD: subclinical organ damage; HT: hypertension; SBP: systolic blood pressure; DBP: diastolic blood pressure; MS: metabolic syndrome; CV: cardiovascular disease.

The 2007 European Guidelines for Management of Arterial Hypertension [13] covered 79% of the total HUNT 2 population, aged 20-89. Figure 3 shows age-standardised prevalence (percentage and absolute numbers) in each risk category, as well as the associated number of follow-up visits recommended per 100,000 adults per year.

As shown in Figure 3, only 6.6% of all individuals with a blood pressure of ≥120/80 mmHg were classified as "average risk". The rest, or 93.4% (i.e., 74% of the total, adult population), were classified as eligible for regular clinical attention and/or drug treatment in the near future, based on their total CVD risk profile, according to guideline recommendations. In the subgroup aged 50-64, the proportion eligible for clinical attention reached 99%.

Implementing the aforementioned model of clinical follow-up visits to our population of 65,028 adults, we found that 296,624 visits per 100,000 adults would be needed per year (Figure 3). This means that 99 GPs per 100,000 adults would be needed in Nord-Trøndelag County to implement these hypertension guidelines. This figure can be compared with the estimated number of 87 GPs per 100,000 adults, who in 2007 served the adult population in the county for all contact reasons.

If individuals in the higher risk categories and those under specific lifestyle supervision were to be seen 3.0 times or alternatively 4.0 times yearly instead of 3.5 times, as previously discussed, the total number of visits would be 260,035 or alternatively 333,212 visits per year. This corresponds to 87 or 111 GP positions, respectively.

**Figure 4:**
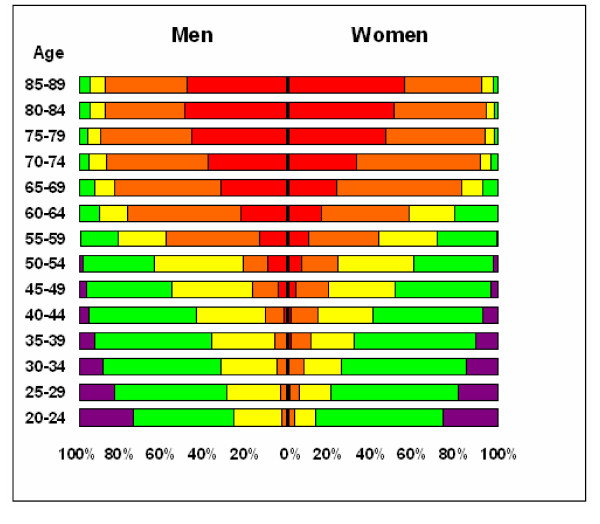
**Gender-specific proportions of individuals within 5-year age groups, labelled at different risk levels**. Gender-specific proportions of individuals within 5-year age groups, labelled at different risk levels according to the 2007 Guidelines for the Management of Arterial Hypertension [13]: average risk (purple), low added risk (green), moderate added risk (yellow), high added risk (orange), and very high added risk (red).

Figure 4 shows the proportion of individuals at different risk levels by age and gender. As expected, the proportion of individuals at higher risk increases with age for both men and women.

## Discussion

Modelling the implementation of current European guidelines on arterial hypertension [13] on a general population of Norwegian adults, aged 20-89, we found that 93.4% of all individuals with blood pressure of ≥120/80 mmHg (i.e., 74% of the total, adult population) would be eligible for regular clinical attention and/or drug treatment, based on their total CVD risk profile. In terms of the primary care workforce, a larger number of GPs would be needed for the sole purpose of implementing the hypertension guidelines, than the number of doctors who currently serve all primary care needs of this population - which is affluent as well as long-lived and healthy-living, by international comparison. These findings raise important questions related to the scientific validity, clinical sustainability and social responsibility of the guidelines.

Some limitations and other methodological considerations related to our implementation model have to be taken into consideration. Compared with other European regions, including regions involved in the MONICA project (third phase, 1992-94) [37], HUNT 2 did not differ significantly with respect to cholesterol levels and smoking habits at the time of data collection. The blood pressure levels, however, were somewhat higher in the HUNT 2 population than in most comparable countries, yet lower than in Finland [37,38].

Our sensitivity analysis of 3.0 and 4.0 follow-up visits instead of 3.5 for those in the higher risk levels and those with lifestyle changes shows that our concerns remain valid, even if the conservative estimate is chosen.

It would obviously have been of interest to qualify the total workload in terms of 'additional preventive measures' as opposed to 'already established workload related to clinical disease'. Our data are however not suited to make valid and transparent calculations of these sub-categories of workload. For instance, we know that a good deal of blood pressure follow-up in Norway takes place in consultations taking place for other contact reasons.

It may be argued that follow-up of known CVD risk patients may demand less, or alternatively more, than the average consultation time. The guideline authors emphasise that blood pressure control is a demanding, clinical task: "Indeed, health providers sometimes wrongly consider the management of hypertension as the matter of few minute visits and reimburse doctors accordingly" [13]. In the presence of doubt, we chose to base our calculations on the average Norwegian GP patient turnover rate. These calculations are however transparent and can easily be adapted to fit healthcare models with higher GP turnover rates or, alternatively, more contact with auxiliary staff and fewer doctor visits.

The aforementioned adjustments made to accommodate the nature of the HUNT 2 data as well as the exclusion of visits related to initial diagnosis and drug titration, will all tend to underestimate the population-at-risk and clinical workload. This, however, does not mean that our final results represent an underestimate. As said, the average blood pressure in the HUNT 2 population was slightly higher than in comparable countries, and the use of ten-year-old population data in our model may also imply a tendency to overestimation as blood pressure levels in the Norwegian population may have decreased since 1995-7. Such trends have at least been observed in some other European regions [39,40]. But even if our model were to overestimate the population-at-risk somewhat, important theoretical, practical and ethical issues need to be addressed.

One crucial question that is hard to answer, and which is not specific for the 2007 hypertension guidelines, is whether the guideline's recommended approach would prove clinically effective if implemented in the general population, just as recommended. We have previously demonstrated how the 2003 European CVD prevention guidelines inflated the high-risk group, most likely due to a phenomenon called retrospective risk bias [17,20,21,24,41], resulting from the fact that mortality from CVD has decreased steadily in Western Europe during recent decades [42]. The reasons for this decline are complex and cannot be accounted for by changes in conventional risk factors and medical interventions alone.

Recently, a prestigious, Norwegian study was conducted on evidence-based implementation of a CVD preventive guideline in general practice [43,44]. It turned out that even motivated GPs receiving tailored information, prompting and feedback showed surprisingly low concordance with the recommendations. This finding accords well with previous studies in national and international settings [45,46]. The lack of adherence, as is usually the case, was interpreted as proof that practicing clinicians are not 'good enough'. This interpretation may however be unsatisfactory. An alternative, or additional, interpretation is that contemporary CVD prevention *guidelines *are not good enough, in the sense that they are not in reasonable concordance with human nature and the realities of clinical practice [47].

The 2007 guideline's evidence-base contains 825 references. None of these discuss how medical professionals may address societal, political, work-related and relational factors, which have all been documented to play significant roles in CVD aetiology and prognosis [47,48]. We realise that it would be a challenging task to accommodate such perspectives in clinical guidelines, but ignoring evidence because it fits poorly with the mainstream, established biomedical understanding of hypertension is neither scientifically nor morally defendable.

## Conclusion

Our findings indicate that the 2007 European blood pressure guidelines have an inherent potential to destabilise the healthcare system in Norway, one of the world's most long- and healthy-living nations, by international comparison. In our view, such a large-scale, preventive medical enterprise can only be regarded as scientifically sound and truly evidence-based, as long as issues of practical feasibility and sustainability are made transparent and discussed [45].

## Competing interests

The authors declare that they have no competing interests.

## Authors' contributions

JAS and LG conceived the study idea. HP analysed the data and wrote the first draft. All authors (HP, LG, JAS, and IH) contributed to interpretation and discussion of the findings. All authors participated in further revisions of the paper and approved the final version.

## Pre-publication history

The pre-publication history for this paper can be accessed here:


